# Association between health literacy, general psychological factors, and adherence to medical treatment among Danes aged 50–80 years

**DOI:** 10.1186/s12877-021-02339-y

**Published:** 2021-06-26

**Authors:** Subash Thapa, Jesper B Nielsen

**Affiliations:** Research Unit of General Practice, University of Southern Denmark, J.B. Winsløws Vej 9, 5000 Odense, Denmark

**Keywords:** Chronic disease management, Health literacy, Medication behavior, Medication perception, Patient self-management, Self-assessed health, Treatment adherence

## Abstract

**Background:**

Understanding behavioral factors associated with low health literacy (HL) is relevant for health care providers to better support their patients’ health and adherence to preventive treatment. In this study, we aim to study associations between low HL and socio-demographic characteristics, medication-related perceptions and experience, as well as general psychological factors among patients aged 50–80 years.

**Methods:**

We used a cross-sectional survey design based on a representative group of 6,871 Danish citizens aged 50–80 years returning a web-based questionnaire with socio-demographic data added from a national registry. Chi-square tests were conducted to analyze associations between low HL and daily use of medication and self-rated health. Chi-square tests and binary logistic regression were conducted for analyzing data from respondents using prescribed medicines daily (*N* = 4,091).

**Results:**

Respondents with low HL were more often on daily medications (19 % [777/4,091] vs. 16 % [436/2,775]; *P* < 0.001) and were more likely to have poorer self-rated health (*P* < 0.001). Among patients on daily medications, low HL was significantly higher among men and those with lower educational attainment and lower family income. Low HL was independently and positively associated with perceptions that taking prescribed medicines daily is difficult and time-consuming, with forgetting to take prescribed medicines, and with lower satisfaction with life and poor self-assessed health.

**Conclusions:**

Our study provides information that patients aged 50–80 years with low HL are challenged on their adherence to treatment plans which is not only related to traditional sociodemographic factors but also on perceptions related to taking medication per se.

## Background

Health literacy (HL) entails ‘’people’s ability to access, understand, appraise, and apply health information to make decisions in everyday life concerning health care, disease prevention and health promotion to maintain or improve their quality of life’’ [1]. Self-management strategies, such as taking medications, for successful prevention and management of chronic diseases often require skills to look for and understand essential health information and interact with healthcare professionals [2]. Low HL levels in this regard is a common challenge shared by many patients globally [3]. In Europe, on average, 1 in 2 individuals cannot understand essential health information, while slightly lower rates of inadequate HL have been noted in the countries to the north, including Denmark, with around 1 in 5 individuals lacking the ability to understand essential health information [3, 4].

In the last two decades, the number of studies on predictors of HL has increased tremendously, with the majority reporting the association of low HL with poor socioeconomic conditions (e.g., poor educational attainment) as well as poorer health outcomes [1, 3, 5–7]. In Denmark, several studies have investigated the association of low HL mainly with poorer health outcomes, e.g. health care seeking or cancer screening behaviors, in specific patient groups [8–14]. However, only a few studies have investigated HL as an outcome variable, and there exists a general agreement in these studies on the association of low HL with lower education and income, unhealthy behaviors (e.g., smoking habit, alcohol consumption) and (actual as well as perceived) poorer health status [4, 12, 13].

Patients aged 50 years and older are more likely to have chronic diseases and in need of long-term self-care and use of preventive medications [15]. Particularly, those with low HL more often have poorer health conditions and have difficulties integrating self-management practices (e.g., taking the prescribed medicines) into their daily routines [5, 16]. This population group, however, is not often well represented in HL studies. Further, the studies that exclusively target elderly individuals often lack the comparability due to small sample size, and/or heterogeneity in measurement tools used or outcomes measured or patient groups targeted [5, 12, 13, 17]. Moreover, in these studies, the association of low HL with medication-related perceptions and experience, and general psychological factors, such as willingness to take health risks and satisfaction with health and life, was not examined.

The association of poor socio-economic characteristics with low HL is plausible and well-described, while it is not clearly understood whether medication-related perceptions and experiences as well as general psychological factors would be associated with low HL among people aged 50 years and older. Such information may add to the existing knowledge base for health care providers to better support their patients’ health and informational needs [2, 18]. Moreover, a larger and more representative sample, would allow identification of relevant patient characteristics in subgroups with low HL [18]. In this study, we aim to investigate the association of HL with socio-demographic characteristics, perceptions and experience of using daily medications as well as with general psychological factors within a representative sample of Danish citizens aged 50–80 years on daily medication.

## Methods

### Sample and Procedure

We used a cross-sectional survey design based on a representative sample of 15,072 Danish citizens aged 50–80 years who were randomly selected and contacted through the national digital mailbox (e-Boks.dk) in 2019. Besides the intended age selection, and the online connection required for access to their e-Boks, no other inclusion or exclusion criteria were used.

Data were collected through a web-based standardized questionnaire administered by Statistics Denmark, and socio-demographic data were added from a national registry. Two reminders were sent through the digital mail. Among the net sample, 6871 persons (45.59 %) returned a completed questionnaire and among those who returned the questionnaire, 4091 persons (59.54 %) reported to use prescribed medications on a daily basis.

### Measurement

The outcome variable was “HL”. Several conceptual models and measurement tools of various domains of HL have been proposed, however most of them have limited applicability due to their focus on a one-dimensional concept of HL (e.g., reading ability and numeracy skill) or on one specific disease condition or one health care setting [18, 19]. A previous Danish study from 2017 measured only 2 subscales of the full HL Questionnaire, which were “understanding health information” and “ability to actively engage with healthcare providers” [14]. In a systematic review, Chesser et al. [5] suggested that there exists no validated HL tool or widely agreed-upon framework to use in an older segment of the patient population in relation to self-management of chronic diseases. In this view, we used a shorter version of HL scale including one item for each 4 important aspects of HL within chronic disease management, as suggested by Poureslami et al. [18]. The scale included four Likert scale questions about: (a) finding information about diseases, (b) finding professional help when ill, (c) a good understanding when communicating with physicians and (d) a good understanding on how to use medicines. All items were presented with 4-point scales (1 = very easy to 4 = very difficult). Item responses were summed up and the sum scores were dichotomized as “non-adequate” (9–16 points) and “adequate” (< 9 points). The cut-off was chosen to reflect the situation in Denmark, where approximately 20 % of the population have previously been rated as having limited HL [4]. Cronbach’s alpha for the 4-item HL scale was 0.83.

The independent variables included socio-demographic characteristics, smoking habit and alcohol consumption, information about prescribed medicines, perceived importance of taking daily medications, perception that taking medications every day is difficult and time consuming, often forgetting to take prescribed medications, and general psychological factors, namely satisfaction with life and health, self-assessed health and willingness to take health risks. The final analysis was conducted among respondents on a daily prescription medication, excluding vitamins, minerals, omega-3 fatty acids, herbal medicines, and other equivalent products.

Socio-demographic characteristics included gender (“male”, and “female”), age (“50–60”, “61–70”, and “71–80”), educational attainment (“basic school’’, “high school/vocational education”, “medium education”, and “higher education”), and average family income per year(“<€33,334”, “€33,334–€46,666”, “>€46,667”). Smoking habits were assessed with a single item: “Do you smoke?”. The variable was coded with three levels: “never a smoker”, “quit smoking”, and “smoker”, and alcohol consumption was assessed with a single item “How many units (equivalent to one glass of wine) of alcohol do you drink usually in a week?”, and answers were categorized into “non-drinker”, “1–14 units” and “>14 units”.

Information about prescribed medicines was measured based on three Likert-scale questions: (a) sufficient information about why to take the prescribed medicine, (b) sufficient information about how to take the prescribed medicine, and (c) sufficient information about potential side effects of the prescribed medicine. All items were presented with 6-point scales (1 = strongly disagree to 6 = strongly agree). Item responses were summed up and the sum scores were dichotomized as “poor” (< 13 points) or “sufficient” (13–18 points). Cronbach’s alpha for the 3-item scale was 0.74.

To understand perceptions and experience of taking prescribed medications, respondents were asked to rate their agreement with “It is difficult and time consuming to take a medication daily’’; and “It is important to take my medication’’; and “I often forget to take my medicine”. All three variables were originally answered based on a 6-point Likert scale (1 = strongly disagree to 6 = strongly agree), and later dichotomized to “disagree” (1–3) and “agree” (4–6).

The general psychological variables included satisfaction with life and health, self-assessed health, and willingness to take health risks. Satisfaction with life was assessed with the single item, “How satisfied are you all in all with your life?” and satisfaction with health was assessed with a single item, “How satisfied are you with your health?” Both questions were presented with response scales from 0 (lowest level of satisfaction) to 10 (highest level of satisfaction), which were subsequently categorized into “low’’ (0–4), “moderate” (5–7), and “strong’’ (8–10). Self-assessed health was based on the single question: “How would you rate your current state of health?” which was rated on a 5-point Likert scale (1 = nearly perfect to 5 = very poor), and was later dichotomized into “good” (1–3) and “poor” (4–5) [20]. Further, willingness to take health risks was assessed by the question, “How do you evaluate your willingness to take a risk related to your health situation?” Participants could answer on a scale from 0 (no risk willingness) to 10 (high risk willingness), and for the analyses, the scores were categorized as “low” (0–4), “moderate” (5–6) and “high” (7–10) [21].

### Statistical analyses

Analyses were performed using Stata 16.0 (StataCorp LP, College Station, TX). Chi-square tests were conducted for the total respondents to analyze association of low HL with daily use of medication and self-rated health. Chi-square tests and binary logistic regression were conducted for the respondents on daily medications to examine the association of low HL with socio-demographic characteristics, perceptions and experience of taking medications as well as general psychological factors. A stepwise approach with a *p*-value < 0.20 for variable inclusion was performed. Significance levels for testing individual variables were set at *p*-value < 0.05.

## Results

Among all respondents (*n* = 6,871), nearly 18 % had low HL and 60 % reported taking prescribed medicines daily (Table 1). Further, respondents with low HL were more often on daily medications (*P *< 0.001) and more likely to have poorer self-rated health (*P* < 0.001).

**Table 1:** Taking prescribed medications daily, self-assessed health and health literacy status of the total respondents

	Health literacy status of the total respondents			
	Non-adequate	Adequate	Total	Chi-square
	n (%)	n (%)	n (%)	*p* value
**Taking prescribed medications daily**				
Yes	777 (18.99)	3314 (81.01)	4091 (59.58)	**< 0.001**
No	436 (15.71)	2339 (84.29)	2775 (40.42)	
Total	1213 (17.67)	5653 (82.33)	6866 (100.00)	
**Self-assessed health**				
Excellent	67 (8.86)	689 (91.14)	756 (11.00)	**< 0.001**
Very good	326 (12.08)	2372(87.92)	2698 (39.27)	
Good	487 (20.66)	1870 (79.34)	2357 (34.30)	
Poor	263 (29.52)	628 (70.48)	891 (12.97)	
Very poor	71 (42.01)	98 (57.99)	169 (2.46)	
Total	1214 (17.67)	5657 (82.33)	6871 (100.00)	

The bivariate analysis within daily users of prescribed medicine (Table 2) found that low HL was significantly more frequent among those having lower educational attainment and lower family income. Further, low HL was significantly more frequent among those who perceived that taking medication daily was difficult and time-consuming, who reported poor information about the prescribed medicines, who often forgot to take their medications, who had lower satisfaction with life and who reported poorer self-assessed health.

**Table 2:** Bivariate analysis of factors associated with health literacy among respondents on daily medications

Variables	Health literacy status of respondents on daily medications			
	Adequate (*N* = 3314)	Non-adequate (*N* = 777)	Total (*N* = 4091)	Chi-square
	n (%)	n (%)	n (%)	*p* value
**Gender**				
Male	1543 (80.11)	383 (19.89)	1926 (47.08)	0.170
Female	1771 (81.80)	394 (18.20)	2165 (52.92)	
**Age-group**				
50–60	976 (80.46)	237 (19.54)	1213 (29.65)	0.302
61–70	1301 (82.19)	282 (17.81)	1583 (38.69)	
71–80	1037 (80.08)	258 (19.92)	1295 (31.65)	
**Highest completed education**				
Basic school	645 (72.47)	245 (27.53)	890 (21.76)	**< 0.001**
High school & vocational	1648 (79.88)	415 (20.12)	2063 (50.43)	
Medium education	727 (88.55)	94 (11.45)	821 (20.07)	
Higher education	294 (92.74)	23 (7.26)	317 (7.75)	
**Average family income per year (€)**				
< 33,334	1398 (76.60)	427 (23.40)	1825 (44.61)	**< 0.001**
33,334–46,666	995 (82.71)	208 (17.29)	1203 (29.41)	
> 46,666	921 (86.64)	142 (13.36)	1063 (25.98)	
**Smoking**				
Never a smoker	1869 (82.08)	408 (17.92)	2277 (58.54)	0.131
Quit smoking	942 (79.97)	236 (20.03)	1178 (28.79)	
Smoker	503 (79.09)	133 (20.91)	636 (15.55)	
**Alcohol consumption** (unit per week)				
Non-drinker	827 (76.36)	256 (23.64)	1083 (26.48)	**< 0.001**
1–14 units	2056 (82.14)	447 (17.86)	2503 (61.20)	
>14 units	430 (85.32)	74 (14.68)	504 (12.32)	
**Information about the prescribed medicines**				
Poor	189 (56.59)	145 (43.41)	334 (8.23)	**< 0.001**
Sufficient	3099(83.19)	626 (16.81)	3725 (91.77)	
**Perception that taking medicines daily is difficult and time consuming**				
Disagree	2669 (83.69)	520 (16.31)	3189 (78.18)	**< 0.001**
Agree	636 (71.46)	254 (28.54)	890 (21.82)	
**Perceived importance of taking medicines**				
Disagree	228 (80.57)	55 (19.43)	283 (7.08)	0.822
Agree	3014 (81.11)	702 (18.89)	3716 (92.92)	
**Often forgetting to take prescribed medicines**				
Disagree	3013 (82.73)	629 (17.27)	3642 (89.37)	**< 0.001**
Agree	289 (66.74)	144 (33.26)	433 (10.63)	
**Satisfaction with life**				
Low	139 (62.05)	85 (37.95)	224 (5.48)	**< 0.001**
Moderate	656 (72.73)	246 (27.27)	902 (22.05)	
Strong	2518 (84.95)	446 (15.05)	2964 (72.47)	
**Satisfaction with health**				
Low	543 (69.26)	241 (30.74)	784 (19.16)	**< 0.001**
Moderate	1121 (79.39)	291 (20.61)	1412 (34.51)	
Strong	1650 (87.07)	245 (12.93)	1895 (46.32)	
**Self-assessed health**				
Poor	625 (69.06)	280 (30.94)	905 (22.13)	**< 0.001**
Good	2689 (84.43)	496 (15.57)	3185 (77.87)	
**Willingness to take health risks**				
Low	855 (78.66)	232 (21.34)	1087 (26.63)	**< 0.001**
Moderate	1625 (80.85)	385 (19.15)	2010 (49.24)	
Strong	826 (83.86)	159 (16.14)	985 (24.13)	

The multivariable analysis (Table 3) found that low HL was significantly more frequent among men, those with lower education attainment and lower family income. Moreover, low HL was independently and positively associated with perceptions that taking prescribed medicines daily is difficult and time consuming, with often forgetting to take prescribed medicines, with lower satisfaction with life and with poor self-assessed health.

**Table 3:** Multivariable analysis of factors associated with health literacy among respondents on daily medications (*N* = 4091)

Variables	B	Wald χ2	*P* value	OR (95 % CI)
**Gender**				
Male				1
Female	0.18	4.49	**0.034**	**1.20 (1.01–1.43)**
**Highest completed education**				
Basic school				1
High school & vocational	0.27	7.78	**0.005**	**1.32 (1.08–1.61)**
Medium education	0.85	35.76	**< 0.001**	**2.35 (1.77–3.10)**
Higher education	1.20	24.50	**< 0.001**	**3.34 (2.07–5.39)**
**Average family income per year (€)**				
< 33,334				1
33,334–46,666	0.15	2.28	0.131	1.16 (0.95–1.42)
> 46,666	0.28	5.80	**0.016**	**1.32 (1.05–1.66)**
**Smoking**				
Never a smoker				1
Quit smoking	-0.07	0.54	0.460	0.93 (0.76–1.12)
Smoker	0.14	1.29	0.253	1.15 (0.90–1.46)
**Alcohol consumption** (unit per week)				
Non-drinker				1
1–14 units	0.03	0.09	0.756	1.03 (0.84–1.25)
>14 units	0.28	3.06	0.081	1.33 (0.96–1.83)
**Information about the prescribed medicines**				
Poor				1
Sufficient	1.11	77.44	**< 0.001**	**3.05 (2.38–3.91)**
**Perception that taking medicines daily is difficult and time consuming**				
Disagree				1
Agree	-0.33	11.15	**0.001**	**0.71 (0.59–0.87)**
**Often forgetting to take prescribed medicines**				
Disagree				1
Agree	-0.57	21.62	**< 0.001**	**0.56 (0.44–0.71)**
**Satisfaction with life**				
Low				1
Moderate	0.20	1.39	0.236	1.23 (0.87–1.73)
Strong	0.57	9.98	**0.002**	**1.78 (1.24–2.55)**
**Satisfaction with health**				
Low				1
Moderate	0.05	0.12	0.718	1.05 (0.80–1.38)
Strong	0.29	3.09	0.078	1.33 (0.96–1.84)
**Self-assessed health**				
Poor				1
Good	0.29	5.24	**0.022**	**1.34 (1.04–1.73)**
**Willingness to take health risks**				
Low				1
Moderate	0.001	0.0001	0.988	1.00 (0.82–1.22)
Strong	0.17	2.01	0.156	1.19 (0.93–1.51)

## Discussion

Low HL was significantly more common among people who were on prescribed medications (19 %) than those who were not (16 %). A previous Danish study by Friis et al. also noted people with long-term conditions have more difficulties in understanding health information as well as in engaging with healthcare providers than the general population [12]. Data on prescribed medicines can be a proxy to having one or more long-term health conditions [22], which is why there is consistency in the given finding between Friis et al.’s study [12] and the present study, regardless of different age-groups targeted between the two studies.

Age was indeed not associated with low HL levels in the present study, which is in contrast to several studies suggesting a positive relationship between age and low HL, especially in the oldest segment of the population [2, 5, 23]. The diminished HL levels with increasing age is mostly due to cognitive decline which is related to aging or having chronic conditions. However, this is less likely to be observed in the present study as people over 80 years who are more at risk of cognitive decline were not targeted in our study [23, 24].

Regardless of the setting, patient groups targeted or measurement tools used, low level of HL has repeatedly been associated with lower levels of education as well as income [5–7]. Demonstrating the same associations in our study support the construct validity of our measurement scale for HL. Low HL was significantly more frequent among those who reported having poor information about prescribed medications, those who perceived difficulties in taking medications daily, and those who often forgot to take their prescribed medications. These findings are in line with previous studies conducted among elderly individuals in other settings [5, 25].

Previous Danish studies suggest that literacy-related needs of the patients with lower education and income and limited HL are not often considered in the context of (self) management of chronic diseases [9, 10, 12]. Differences exist between countries and health care systems, but often it is the general practitioners that takes responsibility for medication adherence as well as continuity of care among patients on daily medications, and they should be aware of this group of patients with HL challenges. The present findings suggest that an assessment of general HL as well as information and perceptions about prescribed medication is an important step to consider in efforts to improve appropriate adherence among elderly patients. For instance, such information may help tailor patient education and counselling programs, which could effectively improve overall self-management behaviors among the patients with limited HL [18].

Further, our findings suggest that HL is not only associated with socio-economic characteristics or medication-related factors, but also with the way a person perceives his/her health status and life satisfaction. Our observed associations between poor self-assessed health and low HL among patients on daily medications is in consistent with previous Danish studies by Svendsen et al. [13] and Aaby et al. [14]. Further, the association between low HL and poorer life satisfaction particularly among elderly individuals has previously been described in a Slovenian study, although with a much smaller sample size (N = 656) and a more narrowly defined study population [26]. In the present study, we were able to reproduce the Slovenian result, but with a larger sample size allowing for subgroup analyses on patients’ characteristics. These subgroup analyses indicated that subjective well-being, which is due to a good level of self-assessed health as well as life satisfaction, is generally associated with higher HL skills and the willingness to practice self-management, which is why health and informational needs of the patients with poorer subjective well-being should be understood and addressed also in a HL context.

### Strengths and Limitations

Our study has notable strengths, which include recruitment of a large sample size to assess the relationship and allow for valid subgroup analyses. Moreover, our sample population has similar demographic composition to the Danish population in this age group, and our findings is representative for settings with similar socio-demographic and healthcare system characteristics as the Danish. The results however should be interpreted within its limitations including a modest response rate.

A major limitation of the present study is the use of several self- constructed questions including HL, and because of this, one-to-one comparability with previous findings on these topics is more limited. For this reason, future HL studies focusing on patients’ behaviors such as self-management and medication adherence should try to use different validated assessment tools. The consistency with previous studies on associations with income and educational attainment, however, supports the internal validity of the HL measurement tool used in the present study. Due to the cross-sectional design, conclusions about causality and temporality cannot be drawn. Eventually, the study was based on an e-questionnaire filled in at home; and we cannot exclude that some patients received assistance completing the questionnaire. Likewise, we cannot exclude that those with very low HL did not participate at all. Self-reporting bias is possible as our e-survey relied on self-reported information.

## Conclusions

The present findings indicate that patients with low HL are challenged regarding adherence to medicinal treatments, due to perceived difficulty in taking medications on a daily basis and forgetting to take prescribed medicines, as well as perceived lower quality of life.

## Data Availability

The datasets used and/or analyzed during the current study are available from the corresponding author on reasonable request.

## Abbreviations

HL: Health literacy
